# Correction: Personalized risk stratification through attribute matching for clinical decision making in clinical conditions with aspecific symptoms: The example of syncope

**DOI:** 10.1371/journal.pone.0252967

**Published:** 2021-06-03

**Authors:** Monica Solbiati, James V. Quinn, Franca Dipaola, Piergiorgio Duca, Raffaello Furlan, Nicola Montano, Matthew J. Reed, Robert S. Sheldon, Benjamin C. Sun, Andrea Ungar, Giovanni Casazza, Giorgio Costantino, on behalf of the SYNERGI (SYNcope Expert Research Group International)

There is an error in affiliation 4 for authors Franca Dipaola and Raffaello Furlan. The correct affiliation 4 is: Internal Medicine, Humanitas Clinical and Research Center, IRCCS, Humanitas University, Rozzano, Italy.

The following information is missing from the Acknowledgements: This work was partially supported by Università degli Studi di Milano, Piano di Sostegno alla Ricerca—Linea 2–2019
