# Supplementary material for: Personalized risk stratification through attribute matching for clinical decision making in clinical conditions with aspecific symptoms: The example of syncope
Source: PLoS One. 2020 Mar 18;15(3):e0228725. doi: 10.1371/journal.pone.0228725 (PMC7080223; doi:10.1371/journal.pone.0228725)
Supplement: S1 Table — BP: blood pressure; HR: heart rate; ECG: electrocardiogram; ED: Emergency Department; CI: Confidence Interval. (DOCX) [file pone.0228725.s001.docx]

**S1 Table. Example clinical cases with the probabilities predicted by attribute matching and clinical judgement**

| **Case** | **Predicted probabilities** |
| --- | --- |
| Male, age ≥ 65 years  Previous myocardial infarction with preserved left ventricular ejection fraction  Postprandial syncope without prodrome while sitting  BP 160/80 mmHg, HR 80, peripheral oxygen saturation 98%, respiratory rate 14, normal body temperature  ECG: unchanged Q waves in the inferior leads | Attribute matching: 15/3388, 5/15 events, 20% (95% CI 7-45%) 10-day serious adverse events, 1 death  ED physician risk assessment: high |
| Male, age ≥ 45 and < 65 years  Unremarkable past medical history  Syncope while sitting with prodrome (lightheadedness)  BP 110/70 mmHg, HR 58, peripheral oxygen saturation 100%, respiratory rate 14, normal body temperature  ECG: sinus rhythm 54 bpm | Attribute matching: 70/3388, 3/70 events, 4% (95% CI 1-12%) of 10-day serious adverse events, 0 deaths  ED physician risk assessment: intermediate |
| Male, age ≥ 65 years  Affected by arterial hypertension  Syncope without prodrome while standing  BP 110/80 mmHg, HR 62, peripheral oxygen saturation 96%, respiratory rate 12, normal body temperature  ECG: sinus rhythm 54 bpm | Attribute matching: 42/3388, 2/42 events, 5% (95% CI 1-16%) of 10-day serious adverse events, 0 deaths  ED physician risk assessment: intermediate |
| Male, age < 45 year  Unremarkable past medical history  Syncope without prodrome while standing with trauma  BP 100/60 mmHg, HR 90, peripheral oxygen saturation 100%, respiratory rate 16, normal body temperature  ECG: sinus rhythm 65 bpm | Attribute matching: 12/3388, 0/12 events, 0% (95% CI 0-24%) of 10-day serious adverse events, 0 deaths  ED physician risk assessment: intermediate |
| Female, age ≥ 45 and < 65 years  Unremarkable past medical history  Syncope with prodrome while sitting  BP 120/75 mmHg, HR 92, peripheral oxygen saturation 98%  ECG: sinus rhythm 62 bpm | Attribute matching: 84/3388, 3/84 events, 4% (95% CI 1-10%) of 10-day serious adverse events, 0 deaths  ED physician risk assessment: intermediate |
| Male, age < 45 year  Unremarkable past medical history  Syncope without prodrome while standing preceded by a painful stimulus  BP 95/65 mmHg, HR 48, peripheral oxygen saturation 99%, respiratory rate 16  ECG: sinus rhythm 43 bpm with signs of vagal activity | Attribute matching: 34/3388, 2/34 events, 6% (95% CI 2-19%) of 10-day serious adverse events, 0 deaths  ED physician risk assessment: low |
| Male, age ≥ 65 years  Unremarkable past medical history  Syncope without prodrome while standing preceded by shortness of breath  BP 125/80 mmHg, HR 80, peripheral oxygen saturation 100%, respiratory rate 20, normal body temperature  ECG: sinus rhythm 56 bpm | Attribute matching: 42/3388, 2/42 events, 5% (95% CI 1-16) of 10-day serious adverse events, 0 deaths  ED physician risk assessment: high |
| Female, age ≥ 65 years  Affected by atrial fibrillation  Syncope while standing with trauma preceded by shortness of breath, lightheadedness, nausea and vomiting  BP 70/40 mmHg, HR 74, peripheral oxygen saturation 93%, respiratory rate 20, body temperature 37.7°C  ECG: sinus rhythm 84 bpm, negative T waives in V3-V5 | Attribute matching: 6/3388, 1/6 events, 16% (95% CI 3-56) of 10-day serious adverse events, 0 deaths  ED physician risk assessment: high |
| Female, age ≥ 65 years  Previous syncope the year before  Syncope while standing with trauma preceded by chest pain, shortness of breath and lightheadedness  BP 120/70 mmHg, HR 88, peripheral oxygen saturation 97%, respiratory rate 23, normal body temperature  ECG: sinus rhythm 82 bpm, V1 and V2 leads compatible with Brugada pattern | Attribute matching: 6/3388, 0/6 events, 0% (95% CI 0-39) of 10-day serious adverse events, 0 deaths  ED physician risk assessment: high |
| Male, age ≥ 65 years  Previous syncope the year before. Known ischemic cardiomyopathy with reduced ejection fraction, congestive heart failure, pulmonary hypertension, valvular heart disease and arterial hypertension  Syncope while sitting with trauma preceded by lightheadedness  BP 120/70 mmHg, HR 80, peripheral oxygen saturation 91%, respiratory rate 24, normal body temperature  ECG: sinus rhythm 84 bpm, negative T waives in the anterior and lateral leads | Attribute matching: 3/3388, 1/3 events, 33% (95% CI 6-79%) of 10-day serious adverse events, 1 death  ED physician risk assessment: high |

BP: blood pressure; HR: heart rate; ECG: electrocardiogram; ED: Emergency Department; CI: Confidence Interval.
