# Supplementary material for: Personalized risk stratification through attribute matching for clinical decision making in clinical conditions with aspecific symptoms: The example of syncope
Source: PLoS One. 2020 Mar 18;15(3):e0228725. doi: 10.1371/journal.pone.0228725 (PMC7080223; doi:10.1371/journal.pone.0228725)
Supplement: Attachment — Submitted filename: responses to reviewers - New.docx [file pone.0228725.s002.docx]

October 27, 2019

Sandro Pasquali, M.D., Ph.D.

Academic Editor - PLOS ONE

Dear Dr. Pasquali,

Thank you for having considered our manuscript entitled “Personalized risk stratification through attribute matching using syncope as a clinical example” for publication in PLOS ONE (PONE-D-19-19894). We would also like to thank you for the thoughtful comments: we have revised the manuscript accordingly and we believe that this process has improved its quality.

Please find attached a marked-up copy of the manuscript that highlights changes made to the original version and an unmarked version of the revised paper without tracked changes, together with point-by-point responses to the points raised during the review process.

Sincerely yours,

Giovanni Casazza, on behalf of all Co-Authors

Here are our responses to the specific points raised during the review process.

This is a good study with some interesting findings. However it is poorly decribed and reported. The Authors should make any effort to increase the quality of their manuscript. Here are some issues to be addressed.

The study is aimed at investigating a new methodology rather than a clinical condition. The title should be reflect the study aim and reworded. For instance, a possibility is “Personalized risk stratification through attribute matching for clinical decision making in clinical conditions with aspecific symptoms: the example of syncope”.

*We appreciate the editor’s suggestion. We changed the title as suggested.*

The clinical challenge of predicting patient risk in an aspecific condition is well presented in the letter to the Editor. However this remains quite unclear in the abstract. It should be made clear in the abstract that the Authors are investigating a promising methodology for quite generic symptoms and that they picked up syncope as an example.

*We appreciate the opportunity to clarify. We changed the abstract accordingly.*

Introduction. The sentence “While CDTs provide information that would be applicable to the nonspecific patient, they lack useful and precise prediction in subjects with specific clinical presentations or needs.” Is probably inaccurate. Prediction tools can be accurate also on a specific patient, they just need to be informative in terms of variables. For instance CTDs are sometimes very accurate in cancer medicine and they are replacing AJCC TNM staging system as highlighted in the 8th edition of the TMN staging manual.

*We agree with the editor that this statement was inaccurate. We tried to explain better our point of view. The sentence now is the following: “As they are based on models, CDTs are able to predict the risk of any hypothetical patient, even those with a combination of risk factors different from all the patients of the derivation cohort. Therefore, we do not know how the CDT will perform in subjects with specific clinical presentations or needs. Indeed, they lack the ability to provide personalized estimates as required in the era of precision medicine. For example, patients with uncommon diseases are likely not to be correctly risk stratified by CDTs. In addition, the risk estimates of composite outcomes that are usually provided by CDTs cannot always be applied to all patients, as the definition of “acceptable risk” depends on the patient at risk. Hence the need to assess a personalized risk rather than providing a simple binary answer [6].”*

Introduction. Introduction is also likely missing the point. The Authors stated that “Since the well-known limitations of the traditionally derived risk stratification tools in predicting adverse events after syncope”. Are the Authors looking at predicting the cause of a condition that underlie a symptom, that is here syncope?

*We appreciate the opportunity to clarify. We changed the text as follows: Moreover, risk stratification is challenging in conditions (as chest pain, shortness of breath and syncope) presenting with aspecific symptoms that can be the manifestation of many possible underlying diseases. In these cases, decision tools are unlikely to accurately identify all the different adverse events related to the possible etiologies. In syncope, which is a paradigm of the above conditions, the traditionally derived risk stratification tools have failed in predicting adverse events [7–12]. Here, an individualized risk assessment would allow an estimate of not only the probability of a composite endpoint, but rather a detailed risk profile that provides the individual risk of each specific outcome (e.g. arrhythmia or pulmonary embolism).”*

Introduction. It should be stated which is the difference between standard predicting tools and attribute matching. The description about AM is in the method but it seems more appropriate to move it to the Intro to facilitate the reading.

*We appreciate the editor’s suggestion. We moved the description about the AM method and the difference between standard predicting tools and AM in the introduction.*

Introduction. Study hypothesis and aims should be clearly stated. It should be stated that the Authors compared AM prediction and pre-probability defined by an expert clinican. This is quite interesting as the Authors probably expected that a large dataset works as accurately as an expert clinicians though in a more reporducible way when compared to a clinician.

*We appreciate the opportunity to clarify. In the present study, we did not perform a formal comparison between AM and clinical judgement. Indeed, the purpose of the study was to describe how the AM method could work in conditions like syncope. A comparison between AM and clinical judgement would require a much larger reference dataset. We just reported some examples of how it could work in real practice. We tried to explain this in the last paragraph of the methods section.*

Introduction. AM should be spelled out.

*We appreciate the editor’s suggestion. We added spelled out AM as suggested.*

Methods. Time frame of the study is needed.

*We appreciate the editor’s suggestion. We added the study time-frame as required.*

Methods. Statistical methodology for AM should be biefly reported and referenced.

*We appreciate the opportunity to clarify. We now added some details on the AM methodology and a reference in the methods section.*

Methods. Outcome variables and measures not reported.

*We appreciate the opportunity to clarify. We added the outcomes of interest in the methods section.*

Results. Which are the 8 selected variables?

*We appreciate the editor’s suggestion. We moved the list of the 8 selected variables from the methods to the results section.*

Results. ED physician. This should be reported in the method section and criteria for each category described.

*We appreciate the editor’s suggestion. We reported the ED physician role in the methods section. As described, there were no criteria to assess risk, as this was left to the ED physician’s judgement.*

Methods/results. The authors mentioned that AM works better than traditional prediction, which may well be the case. However, in the manuscript such comparison has not been made. Since the Authors have a pretty large dataset they should be able to run this comparison. For instance, they can fit a predictive model based on regression for predicting SAE and test in on their 10 prospective patients. Then they should compare AM and traditional prediction based on regression.

*We appreciate the opportunity to clarify. The purpose of this study was not to make a comparison between AM and the traditional prediction methods. Indeed, we cannot state that AM works better, because a formal comparison would require thousands of patients in the reference database for AM to work. As stated in the study aim, we only wanted to test how AM could work with a real life example and to show that it could allow a different approach.*

Method/results. Are 10 patients enough for this study? Was a sample size calculation performed? If not, which is the reason?

*We would like to thank the editor for this comment. As the 10 patients were only an example and we did no attempt to “validate” the AM method on them, no sample size calculation was performed. If the editor feels that this in confusing rather than useful, we could remove the 10 example patients from the manuscript.*

Discussion. This is a well balanced discussion.

*We appreciate the editor’s comment*
