# Supplementary material for: Personalized risk stratification through attribute matching for clinical decision making in clinical conditions with aspecific symptoms: The example of syncope
Source: PLoS One. 2020 Mar 18;15(3):e0228725. doi: 10.1371/journal.pone.0228725 (PMC7080223; doi:10.1371/journal.pone.0228725)
Supplement: Attachment — Submitted filename: Response to Reviewers.docx [file pone.0228725.s003.docx]

January 5, 2020

Sandro Pasquali, M.D., Ph.D.

Academic Editor - PLOS ONE

Dear Dr. Pasquali,

Thank you for having considered our revised manuscript entitled “Personalized risk stratification through attribute matching for clinical decision making in clinical conditions with aspecific symptoms: the example of syncope” for publication in PLOS ONE (PONE-D-19-19894R1). We would also like to thank you and the reviewer for the thoughtful comments: we have revised the manuscript accordingly and we believe that this process has improved its quality.

Please find attached a marked-up copy of the manuscript that highlights changes made to the original version and an unmarked version of the revised paper without tracked changes, together with point-by-point responses to the points raised during the review process.

Giovanni Casazza, on behalf of all Co-Authors

Here are our responses to the specific points raised during the review process.

Additional Editor Comments (if provided):

Authors have replied to comments raised in the previous review. Although they agreed in principle with the comments, only minor changes have been made throughout the text. More substantial changes are needed to improve the manuscript. The main idea behind this manuscript should be that the presented methodlogy is interesting and promising and a pilot has been conducted to show this. A larger study is clearly needed to validate the method, either looking at syncope or other conditions. In other words, findings are hypothesis generating rather than conclusive. In this regards, authors should make very clear what their next step will be.

*We appreciate the editor’s suggestion. We tried to further clarify that this is a pilot study and that studies on much larger datasets and comparing this method to the traditionally used methods for risk prediction are needed. We added what the next step will be, namely the application of AM to larger datasets through the use of administrative data in conditions in which there is less clinical heterogeneity and to compare AM with the traditional risk stratification tools.*

The manuscript has been sent for additional review and comments of Reviewer#2 which are now available need to be carefully addressed in order to meet requirement for publication in PLOS ONE.

Reviewers' comments:

Reviewer's Responses to Questions

**Comments to the Author**

1. If the authors have adequately addressed your comments raised in a previous round of review and you feel that this manuscript is now acceptable for publication, you may indicate that here to bypass the “Comments to the Author” section, enter your conflict of interest statement in the “Confidential to Editor” section, and submit your "Accept" recommendation.

Reviewer #1: All comments have been addressed

2. Is the manuscript technically sound, and do the data support the conclusions?

The manuscript must describe a technically sound piece of scientific research with data that supports the conclusions. Experiments must have been conducted rigorously, with appropriate controls, replication, and sample sizes. The conclusions must be drawn appropriately based on the data presented.

Reviewer #1: Partly

3. Has the statistical analysis been performed appropriately and rigorously?

Reviewer #1: No

*We appreciate the reviewer’s comment. We have now added logistic regression and updated the methods paragraph.*

4. Have the authors made all data underlying the findings in their manuscript fully available?

The [PLOS Data policy](http://www.plosone.org/static/policies.action#sharing) requires authors to make all data underlying the findings described in their manuscript fully available without restriction, with rare exception (please refer to the Data Availability Statement in the manuscript PDF file). The data should be provided as part of the manuscript or its supporting information, or deposited to a public repository. For example, in addition to summary statistics, the data points behind means, medians and variance measures should be available. If there are restrictions on publicly sharing data—e.g. participant privacy or use of data from a third party—those must be specified.

Reviewer #1: No

*We have indicated that data from this study are available upon request. The database analyzed during the current study involves individual-level patients’ data from 5 studies (Costantino G – JACC 2008; Reed MJ – JACC 2010; Del Rosso A – Heart 2008; Quinn JV – Ann Emerg Med 2004; Sun BC – Ann Emerg Med 2007). Public access to the 5 databases is closed and permission to use data for this study had been obtained from each participating center in occasion of the database creation (Costantino G, Am J Med 2014;127:1126.e13-25). Therefore, the authors of the current paper do not have the permission to share de-identified data, which should be asked to the IRB of each corresponding author’s institution.*

5. Is the manuscript presented in an intelligible fashion and written in standard English?

PLOS ONE does not copyedit accepted manuscripts, so the language in submitted articles must be clear, correct, and unambiguous. Any typographical or grammatical errors should be corrected at revision, so please note any specific errors here.

Reviewer #1: Yes

6. Review Comments to the Author

Please use the space provided to explain your answers to the questions above. You may also include additional comments for the author, including concerns about dual publication, research ethics, or publication ethics. (Please upload your review as an attachment if it exceeds 20,000 characters)

Reviewer #1: The authors quote the following sentence in the conclusions: "our study shows that the AM method could be used to predict the risk of adverse events in clinical practice". However, the paper makes no systematic comparison with the state of the art to support this view. Without this comparison the paper turns out to be nothing more than an exercise in style.
Several times the authors have been asked for this by the reviewers, with an answer "we did not perform a formal comparison between AM and clinical judgement. Indeed, the purpose of the study was to describe how the AM method could work in conditions like syncope" and "The purpose of this study was not to make a comparison between AM and the traditional prediction methods. Indeed, we cannot state that AM works better, because a formal comparison would require thousands of patients in the reference database for AM to work. As stated in the study aim, we only wanted to test how AM could work with a real life example and to show that it could allow a different approach". These answers, from my personal point of view, indicate a lack of attention in the normal scientific approach in which, if a method is to be shown, all the specifications for comparison with the other methods must also be provided.

*We appreciate the opportunity to clarify. As it is the first study on the use of AM in the context of decision making in clinical conditions with aspecific symptoms, this study is not intended to show that AM works better or worse than logistic regression or clinical judgement, but only to assess its potential applications. Indeed, the intrinsic characteristics of AM and regression make it unlikely that a method based on data such as AM could work better than a method based on a model derived from a small dataset. Therefore, we felt that a systematic comparison with the state of the art would not be appropriate at this stage. However, as the editor and the reviewers deemed it important, we added a comparison with a model based on logistic regression and we are open to any suggestions to improve such comparison.*

Please, provide:
- variable selection criterion and cut-points justification

*We appreciate the opportunity to clarify. The database we used was collected for different purposes and we used as predictors the eight variables in common between the original datasets with no a priori decision on the number of predictors to be selected. We acknowledged this in the limitations of the study.*

- prediction accuracy measure, in order to enable the reader to judge the method.

*We appreciate the reviewer’s suggestion. We added an assessment of the overall diagnostic performance of both multivariate logistic regression and AM with ROC curves and their AUC.*
